# Effect of Hele–Shaw cell gap on radial viscous fingering

**DOI:** 10.1038/s41598-022-22769-x

**Published:** 2022-11-08

**Authors:** Sada Nand, Vandita Sharma, Santanu Kumar Das, Srikant Sekhar Padhee, Manoranjan Mishra

**Affiliations:** 1 Department of Mathematics, Indian Institute of Technology Ropar, Rupnagar, Punjab 140001 India; 2 Department of Mechanical Engineering, Indian Institute of Technology Ropar, Rupnagar, Punjab 140001 India

**Keywords:** Fluid dynamics, Computational science, Hydrology

## Abstract

The flow through a Hele–Shaw cell is an experimental prototype to study the flow through a porous medium as well as the flow in microfluidic devices. In context with porous medium flows, it is used to visualize and understand hydrodynamic instabilities like viscous fingering (VF). The gap between the plates of the cell is an important parameter affecting the flow dynamics. However, the effect of the gap on the Hele–Shaw cell flows has been minimally explored. We perform experiments to understand the effect of the gap on VF dynamics. It is observed that a minimum gap is required to observe rigorous fingering instability. The onset time of instability, as well as the width of the fingers, increases with an increment in the gap due to a decrease in the convection. The instability increases with an increase in Péclet number, but the effect of gap width on fingering patterns is evident with broader fingers observed for larger *b*. The results are validated by performing numerical simulations. It is further shown that the gap-averaged three-dimensional simulations using the Stokes law approach and the two-dimensional Darcy’s law result in a small gap Hele–Shaw cell.

## Introduction

A Hele–Shaw cell constitutes two parallel glass plates separated by a narrow space^1–4^. The distance between the two glass plates is referred to as the width of the gap of the cell. The flow through a HS cell with closely spaced glass plates or small gap is mathematically analogous to the flow through a two dimensional porous medium^5^. Also in the experiments, the presence of the glass plates help ease the visualisation of the dynamics which is otherwise difficult in an opaque porous medium. When a more viscous fluid is displaced by a less viscous one in a porous medium, the interface deforms into finger-like patterns, often termed as viscous fingering (VF)^2,6–8^. With many industrial applications including oil recovery processes^9^, fluid mixing^10^, chromatography separation^11^, microdischarges in plasmas^12^, CO$_2$ sequestration^13,14^, flow in granular media^15^ and cell fragmentation in biomechanics^16^, dynamics of VF has been of interest to many researchers. Many experimental studies^1,2,17^ have been conducted to understand VF dynamics. The fingering instabilities are visualised in the laboratory with the help of a HS cell.

Many studies^2,18–22^ have focussed on gaining insights into VF dynamics. Two kinds of displacements, viz., rectilinear and radial are used to understand VF. We term the VF observed using latter displacement of the fluids as radial VF. The velocity of the fluids depend upon the kind of displacement considered. The velocity is uniform for rectilinear while spatially varying for the radial displacement which is a potential flow. Thus, a competition between the forces due to diffusion and convection is evident to affect radial VF dynamics. This competition has been utilised to control VF by considering a finite source^2^ by performing simulations as well as experiments. In another study, the effect of diffusion on VF using rectilinear displacement is studied by Vienne and Marié^21^ using lattice Boltzmann simulations. Similar method has been employed by Shiri and Shiri^23^ to investigate the effects of the flow rate on the instability, in the presence permeability contrast.

A lot of studies have focused on varying the gap of the HS cell^17,24,25^ with an aim to control the instability. Pihler-Puzović et al.^26^ used an elastic membrane at the top wall of the cell and succeeded in suppression of the instability due to increase in gap owing to the deformations in the elastic sheet. Such fluid-structure interactions resulted in the onset of instability being delayed. In another study^17^, a time dependent gap is considered to abate the instability. A tapered HS cell is a type of the HS cell in which the gap is either diverging or converging in the direction of fluid flow. Al-Housseiny and Stone^25^ theoretically reported a means of controlling the instability using a tapered HS cell. They observed the dependence of stability on viscosity contrast and the quotient of Capillary number and depth gradient. Recently, Bongrand and Tsai^24^ explored the effect of depth gradient and flow rates on the VF in a radially tapered HS cell. Using experiments and linear stability analysis, it is established that a converging HS cell can suppress the instability. Thus, varying the gap of the HS cell can control the instability. This hints that even for a non-tapered HS cell, not all gaps can be used to understand and visualise VF dynamics. Therefore, the cell gap has a major impact on VF dynamics, which, to the authors’ awareness, has not been discussed in the literature. In this work, we perform experiments to gain insight into this.

The dynamics of viscous fingering are modelled mathematically using Darcy’s law. Although it is an empirical law, it can be derived from the Navier–Stokes equation. The underlying assumption in the derivation is that the aspect ratio of the HS cell is very less, that is, the gap between the plates is very small compared to the width of the plates. For miscible HS displacements, the Darcy-based modelling technique has had great success in replicating a number of significant empirically observed phenomena. For instance, early studies by Tan and Homsy^18,19^ using linear stability analysis demonstrate that increasing viscosity ratios increase the growth rates and shorten the wavelengths of the most unstable mode which is in line with the experimental results. Further, the tip-splitting phenomenon is successfully reproduced in the subsequent non-linear simulations by Tan and Homsy^27^, and the physical process underlying it is explained. Darcy-based predictions, however, have recently been compared against a number of different strategies. Graf et al.^28^ performed a three dimensional study by considering Stokes equation and conducting a linear stability study, and the results show strong agreement with the growth rates observed in experiments for the Rayleigh number across a five order of magnitude range. The dispersion relations obtained using Stokes equation and similar findings from the gap-averaged Darcy approach have both been compared by these authors, and a satisfactory matching only for moderate values of the Rayleigh numbers is reported. For large values of the non-dimensional parameter Rayleigh number, the three-dimensional effects within the gap become significant, making it difficult to represent the primary physical mechanisms by averaging across the gap. By using a gap-averaged Navier–Stokes–Darcy equation, Martin et al.^29^ explored the same problem and produced dispersion relations for the Rayleigh–Taylor instability that match favourably to three-dimensional lattice BGK simulations. Numerous researchers^30–34^ have discussed additional Stokes-based linear stability results for chemically reacting and variable viscosity HS displacements, demonstrating similar differences between Stokes-based and gap-averaged results.

In the present work, we aim to investigate the effect of the gap on the VF dynamics. Thus, we perform the three dimensional simulations with Stokes equation. Further, we try to gain insight into the fact if the averaged Stokes results match with the two dimensional Darcy results or not? Our objective is to solve the unsteady Stokes equation in three-dimension by varying the gap between the plates and take an average along the gap direction to visualize the dynamics in two-dimension.

## Results

We carry out experiments using a radial HS cell. The objective is to understand (i) the evolution of the fingering patterns with the variation in the gap and (ii) the effect of key parameters like flow rate and viscosity contrast between the two fluids on the instability. Non-linear simulations using finite element based COMSOL Multiphysics®^35^ are performed to validate the experimental observations. Three dimensional simulations are implemented using Stokes equation for the conservation of momentum. A qualitative agreement is observed with the experimental findings. We further attempt to understand the two dimensional effects by averaging the numerical results and comparing with a two dimensional study conducted using Darcy’s law. We first present and discuss the experimental results.

**Figure 1:**
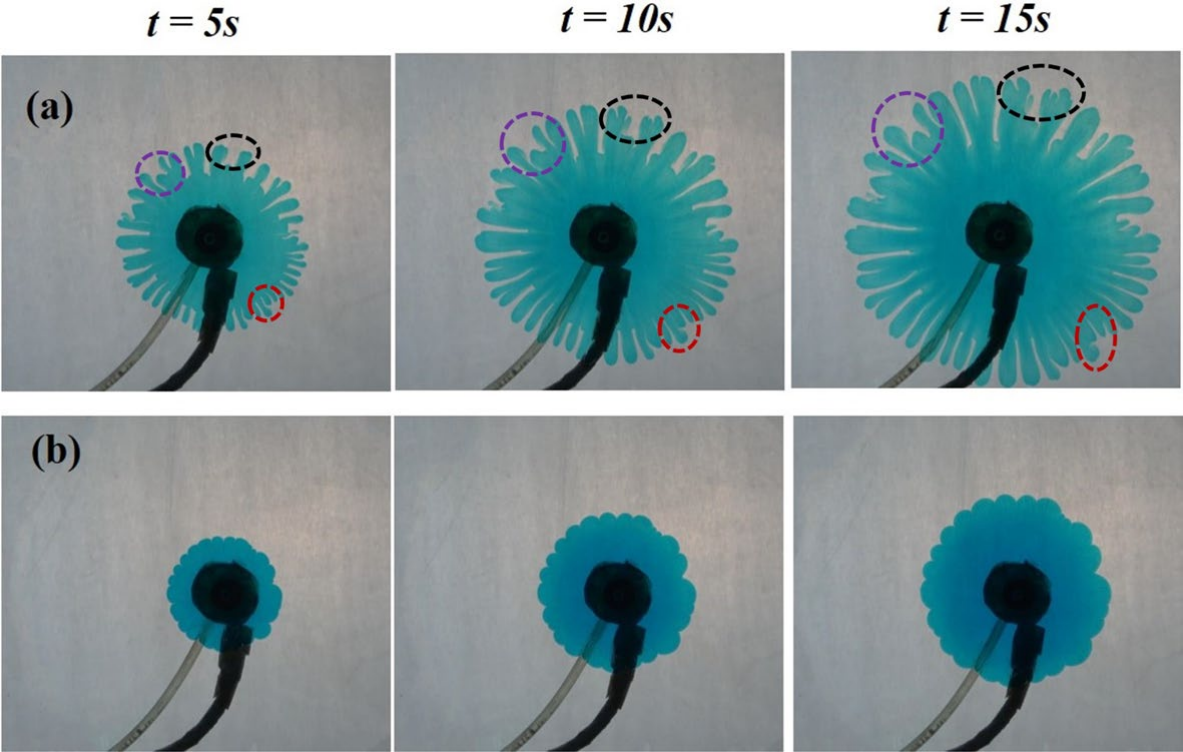
Temporal evolution of the VF dynamics for $M=3, Q=0.1$ mL/s and $b=$ (**a**) 0.3 mm, (**b**) 0.8 mm. An example of tip-splitting, merging and shielding is marked respectively with a black, red and purple circle/ellipse.

### Experimental results

The less viscous fluid is pumped into the HS cell at two constant volume injection rates $Q=0.1 ~\text {mL/s}, 0.2 ~\text {mL/s}$. Only positive log mobility ratios upto $M=3$ are investigated, however, we only show the results for $M=2, 3$ as similar VF dynamics are observed for other values. Different widths viz., $b=0.3, 0.5, 0.8, 1.0 ~\text{mm}$ are used in the experiments. For various combinations of the three parameters (*M*, *Q*, *b*) tested, each experiment was run a minimum of three times to ensure repeatability. We show the snapshots of the top view of the HS cell in the figures. We fix $M=3, Q=0.1 ~\text {mL/s}$ and show the fingering dynamics at different times for two widths of the gap in Fig. 1. VF dynamics features phenomena like shielding, tip-splitting and merging^17,19^. As the name suggests, tip-splitting is when the fingertip divides into two or more fingers. On the other hand, when two or more fingers combine together, it is known as merging. Shielding is referred to as the dynamics when one finger prevents other finger from growing. These features of VF are predominantly visible and marked for $b=0.3$ mm in Fig. 1a, while the dynamics are not rigorous for $b = 0.8$ mm (see Fig. 1b) even for such high viscosity contrast. At any time, the interface is slightly distorted for the larger gap considered. The space between the glass plates of the HS cell is therefore a key aspect in visualising and comprehending the VF instability.

**Figure 2:**
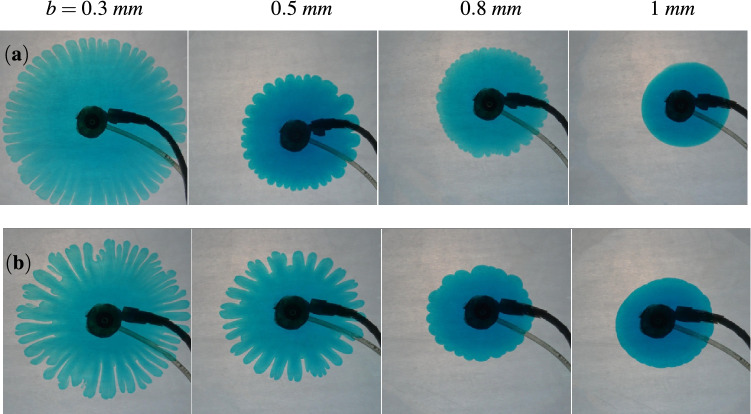
Consequences of changing the gap and the log-mobility ratio. The dynamics are shown at $t=15$ s for $Q=0.1 ~\text {mL/s}$ and (**a**) $M=2$, (**b**) $M=3$. As we move from left to right in each row, the gap increases.

The viscosity contrast is known to be an important factor in determining the fate of the instability^19,25^ . Thus, while maintaining a constant flow rate, we explore the impact of modifying the log-mobility ratio on changing the gap. For a fixed *b*, increasing *M* necessitates a more rigorous instability which is observed in Fig. 2 on comparison of the dynamics in the two rows. The first row is for $M=2$ while the second row is for $M=3$. With an increasing gap, an abated instability is evident as we move from left to right in each row. For $b=1$ mm, no instability is evident for $M=2$, while the front is slightly distorted for $M=3$. Thus, for a larger gap, a sufficiently high viscosity contrast might be required to trigger the instability. Also, it can be inferred that an increase in the gap results in a delayed instability but wider and lesser number of fingers. Additionally, it is clear that the fingers start to show when the value of *b* is decreased, with the most prominent fingers being apparent for the smallest gap taken into account in the Fig. 2.

**Figure 3:**
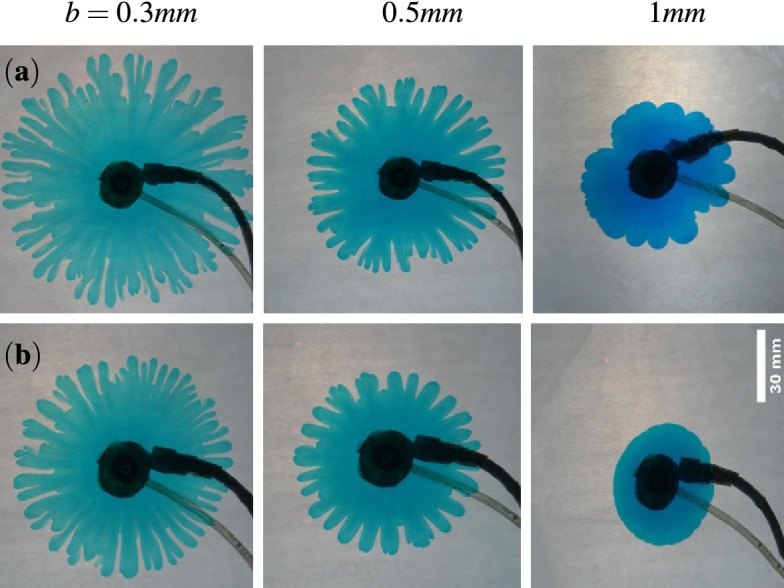
The fingering patterns for $M=3$ at $t=10$ s and different flow rate, (**a**) $Q=0.2 ~\text {mL/s}$, (**b**) $Q=0.1 ~\text {mL/s}$. The instability abates with an increase in the gap from left to right.

So far, for a fixed flow rate, we have seen that the instability decreases when the spacing is increased. It is intuitive to ask what happens with varying the constant flow rate? Does a higher flow rate and thus a faster convection support the instability even for a large gap in contrast to what is observed so far? To gain insight into this, we vary the flow rate and the gap while fixing the log-mobility ratio as $M=3$ in Fig. 3. For a fixed *b*, longer fingers are observed for a larger *Q* as is evident from comparison of VF dynamics in Fig. 3. This is a consequence of the more velocity for a larger flow rate which results in the fluid traveling a larger distance and hence longer fingers. On the contrary, an increase in *b* results in smaller fingers as the area available for the fluid flow increases resulting in a smaller velocity of the fluid. Instability for a high width of $b=1 ~\text {mm}$ for $Q=0.2 ~\text {mL/s}$ in Fig. 3a indicates that a higher flow rate may result in the instability for a larger gap but the instability will always be reduced in comparison to that observed for smaller *b*.

### Quantitative analysis

**Figure 4:**
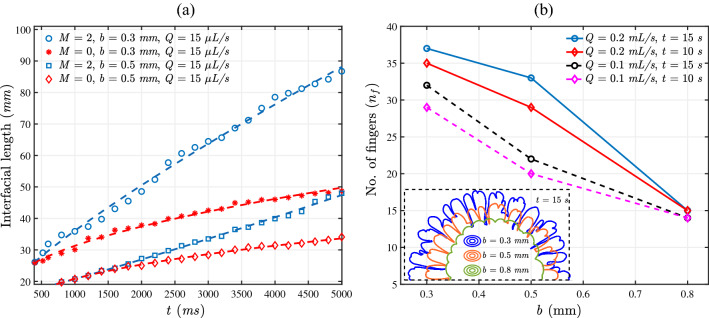
(**a**) Interfacial length versus time for the experiments for various gaps. The interfacial length decreases and the onset time is delayed with increasing gaps. (**b**) Number of fingers as a function of width of the gap for $M=3$. Inset shows the contour for $M=3$ at $t=15$ s for $Q=0.1 ~\text {mL/s}$.

A most used quantitative measure for comparison of the VF dynamics is the length of the pattern measured in terms of the interfacial length^2,36^. A rigorous instability will result in a larger interfacial length. We calculate the interfacial length of the experimental VF patterns in terms of the perimeter using the built-in Wand (tracing) tool in ImageJ. The Wand tool works with detection of the edges. The median filter is used to smoothen the images which are taken as input for interface tracing in the Wand tool. We consider the upper half of the experimental domain to avoid the presence of the flow injection pipes. The processed data is used in MATLAB to plot the interfacial length curve as a function of time. The interfacial length of the patterns is shown in Fig. 4a for various gaps and the log mobility ratios. Even for no viscosity contrast between the fluids, that is, $M=0$, the interfacial length is higher for a smaller *b*. This is due to the larger distance covered by the fluid when the gap between the plates is less. As we consider the viscosity contrast between the fluids ($M\ne 0$), the interfacial length increases. However, it is clear that the interfacial length for $M \ne 0$ reduces as *b* increases signifying an abated instability with an increase in the gap between the plates of the cell.

Further, the interfacial length plots can be used to gain insight into the onset time of instability. The minimum time when the instability begins to appear is termed as the onset time of instability^37^. Before the instability sets in for $M \ne 0$, the interface will grow as a circle similar to the viscosity matched case, that is, $M = 0$. Thus, the interfacial length plots for the fixed *b*, *Q* will be coincident for any value of *M* before the onset of instability. Consequently, the minimum time when the interfacial length curves for $M \ne 0$ deviate from the $M =0$ curve is the onset time of instability. It is evident in Fig. 4a that the onset time for $b=0.3 ~\text {mm}, Q= 15 ~\upmu \text {L/s}$ and $M=2$ is $\sim 500$ ms while the instability sets in at a much later time $\sim 1500$ ms for $b=0.5$ mm. It is therefore concluded that the gap plays significant role in the VF dynamics and the onset is delayed with an increase in the gap of the HS cell. This is in accordance with existing studies with tapered cell involving flow of immiscible fluids^24–26^.

**Figure 5:**
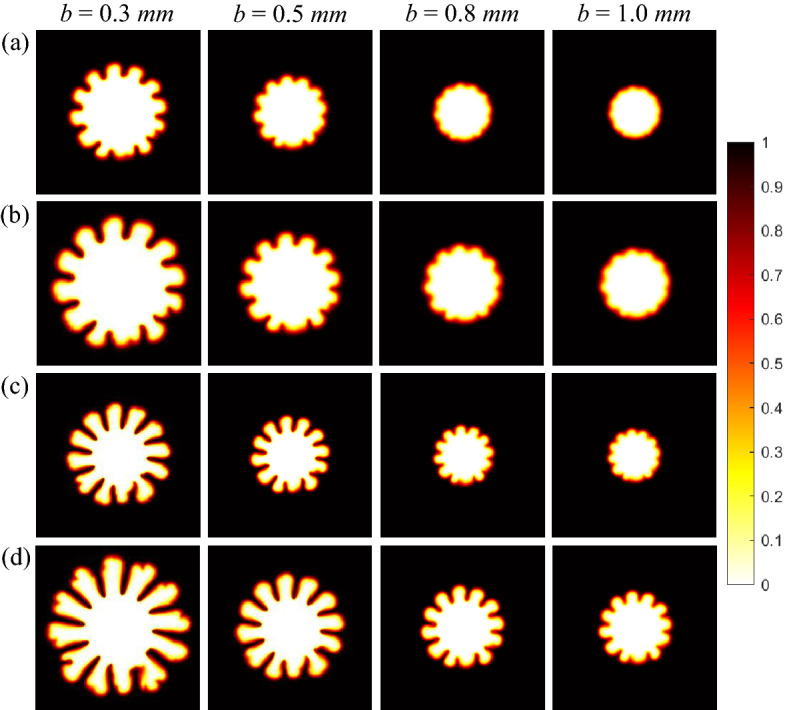
Density plots of the concentration *c* at different gaps, (**a**) $M=2$, $U= 5 ~\text {mm/s}$, (**b**) $M=2$, $U= 10 ~\text {mm/s}$ , (**c**) $M=3$, $U= 5 ~\text {mm/s}$ and (**d**) $M=3$, $U= 10 ~\text {mm/s}$ at $t=15$ s.

**Figure 6:**
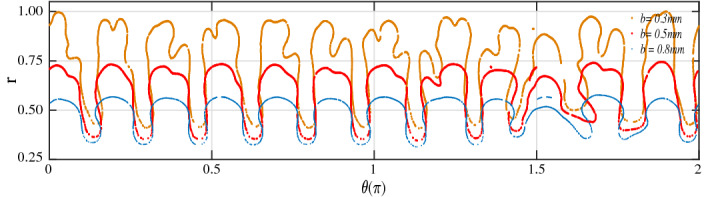
Plots of the 0.5 concentration contour for $M = 3, U = 10 ~\text {mm/s}$ in the polar coordinates ($r,\theta$) at $b = 0.3$ mm, 0.5 mm and 0.8 mm.

To further, quantify the effect of gap on VF dynamics, we calculate the number of the fingers for various gaps and flow rates. Using the *imcontour* command in MATLAB, we plot the contours for various *b*, *Q* at a fixed time (see inset of Fig. 4b) and calculate the number of fingers manually. The number of fingers for $M=3$ are plotted in Fig. 4b. For a fixed flow rate, more fingers are present for a smaller gap at any given time. As seen in Fig. 4b, increased flow rate causes stronger convection, which increases the number of fingers. The length of the fingers is quantified by plotting the contours of the fingering pattern at a fixed time in the inset of Fig. 4b. Clearly, the finger length is more for a smaller *b* supporting the fact that smaller the gap, more rigorous is the instability.

### Numerical results

We perform the numerical study taking into account the three dimensional effects arising due to finite gap of the HS cell. However, we plot the concentration field in Fig. 5 by taking an average along *z* direction. We specify the injection velocity *U* in place of the flow rate *Q*. It is clear in Fig. 5 that for a fixed *M* and *U*, the instability enhances with a decrease in the gap with the most rigorous instability visible for the smallest gap considered. The instability is more emergent with tip splitting visible for the larger viscosity contrast in Fig. 5c in comparison with Fig. 5a . The largest width $b=1$ mm shows instability for the larger *M* in agreement with the experiments.

Further, the effect of the flow rate or equivalently the injection velocity shown in Fig. 5c,d is in agreement with experimental findings reported in this work. The finger length decreases as the gap widens, however, the fingers are longer for the higher injection velocity (flow rate) for a fixed *b*. Thus, the numerical study so far qualitatively validates the experimental investigation.

#### Quantitative analysis

A suppression in fingering instability with increase in gap can be observed in the polar coordinate ($r,\theta$) plots of the concentration contour in Fig. 6. To obtain the plots in the polar coordinates $(r, \theta )$, we extract the (*x*, *y*) coordinates of the concentration from COMSOL. The extracted coordinates are exported to MATLAB and using *imcontour* command, we save the coordinates of the 0.5 concentration contour. Then we make the transformation $r =\sqrt{x^2 + y^2}$ and $\theta = \tan ^{ - 1} ({y/x})$. We looked for the highest *r*-value achieved after computing the *r* values for all the gaps that were taken into consideration. The *r*-values depicted in Fig. 6 are normalised using this maximum value. This normalisation made it easier to compare the finger length for various *b* and kept the maximum value of the *r*-axis in Fig. 6 at 1. Rigorous instability with tip-splitting is evident for $b = 0.3$ mm in Fig. 6, while only toe shaped fingers exist for $b= 0.8$ mm. The length of the fingers for various gaps can be easily compared in the polar coordinate plots. Clearly, longer fingers are evident for the smallest gap considered, in agreement with the experimental findings.

**Figure 7:**
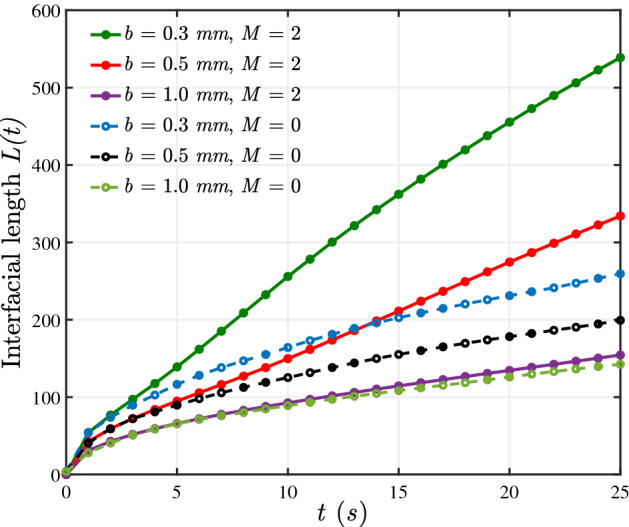
Interfacial length plots for $U= 10 ~\text {mm/s}, M=2$ and $M=0$ for various gaps.

To quantitatively analyse the effects of gap on the fingering patterns, we plot the evolution of normalized interfacial lengths *L*(*t*), given by

1 $$\begin{aligned} L(t)= & {} \frac{1}{L(0)} \int \limits _\Omega | \nabla {c} | ~d\Omega , \end{aligned}$$

2 $$\begin{aligned}= & {} \frac{1}{L(0)}\int \limits _x \int \limits _y \sqrt{\bigg (\frac{\partial c}{\partial x}\bigg )^2+\bigg (\frac{\partial c}{\partial y}\bigg )^2} dy ~dx, \end{aligned}$$

where, *L*(0) is the interfacial length at $t=0$ and is equal to the circumference of the injection hole. The interfacial length for $M=2, U=10 ~\text {mm/s}$ and various gaps is shown in Fig. 7. As *b* is decreased, the interfacial length increases indicating rigorous instability for $M \ne 0$ and a stronger convection for $M=0$. Further, we use the interfacial length plot to quantify the onset of instability as explained in Fig. 4. The minimum time when the interfacial length curve for $M \ne 0$ deviates from the curve for $M =0$ is the onset time of instability. In agreement with the experimental findings, the onset time increases with an increment in the gap.

### Effect of Péclet number

In the numerical as well as experimental discussions, we have presented the results in terms of dimensional quantities, *viz.,* gap width (*b*), flow rate (*Q*) and injection velocity (*U*). A non-dimensional parameter Péclet number (*Pe*) can be defined in terms of these quantities as follows

$$\begin{aligned} Pe_{exp}= \frac{Q}{b D} \text { and } Pe_{num} = \frac{A \times U}{b D}, \end{aligned}$$

where $Pe_{exp}$ and $Pe_{num}$ respectively represent the Péclet number for experiments and the simulations. Here $A=\pi r_0^2$ is the area of the injection hole used in simulations and *D* is the diffusion coefficient of the fluids. Both the definitions of the Péclet number are equivalent but we have utilised two different definitions in order to account for the quantities used in experiments and simulations. The Tables 1 and 2 show various values of the two Péclet numbers used in the present work. It is evident that the order of *Pe* is different in the experiments and numerical simulations. But we also did experiments for $Q=15\, \upmu$L/s as depicted in Fig. 4a. For this value of flow rate, we get $Pe_{exp}=1500, 2500$, respectively for $b=$ 0.5 mm, 0.3 mm, which is of the same order of the $Pe_{num}$ values.

**Table 1:** Values of $Pe_{exp}$.

*b*(mm)	0.3		0.5		0.8		1	
*Q* (mL/s)	0.1	0.2	0.1	0.2	0.1	0.2	0.1	0.2
$Pe_{exp}$	16,667	33,334	10,000	20,000	6250	12500	5000	10,000

**Table 2:** Values of $Pe_{num}$ for numerical simulations.

*b*(mm)	0.24		0.3		0.5		0.8		1	
*U* (mm/s)	5	10	5	10	5	10	5	10	5	10
$Pe_{num}$	1178	2356	942.5	1885	565.5	1131	353.4	706.875	282.75	565.5

**Figure 8:**
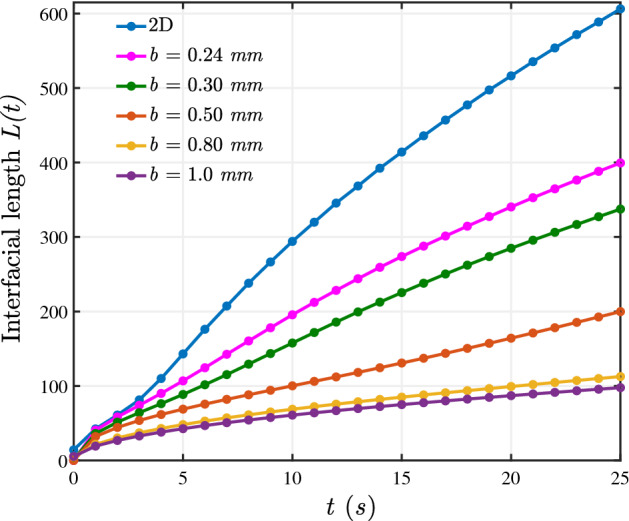
Interfacial length plots for $M=2, U= 5 ~\text {mm/s}$. The 2*D* curve corresponds to the results obtained by solving the Darcy’s law. Clearly, the three dimensional results approach the 2*D* results with a decrease in the gap.

It is known in literature for rectilinear^20^ as well as radial displacement^2^ that the instability becomes rigorous with an increase in *Pe*. In the present work, increase in *Pe* is equivalent to (i) a decrease in gap width for fixed flow rate or (ii) to an increase in flow rate (or injection velocity) keeping *b* fixed. It is evident in Fig. 3 that the instability becomes rigorous with an increase in *Pe*. Comparing the dynamics across Fig. 3a indicates that the instability is abated with a decrease in *Pe* on account of increase in *b*. Further, a comparison in a column in Fig. 3a,b again supports the fact that instability increases with an increase in *Pe*. This is a consequence of increasing flow rate. Interestingly, the effect of gap width is evident when we compare the dynamics for same *Pe* but different *b*. Figure 3a for $b=1$ mm and Fig. 3b for $b=0.5$ mm correspond to $Pe=10,000$. It is visible that the instability is present in both cases but the fingers are broader and witness no VF mechanism for larger *b*. Similar conslusions can be drawn from numerical results in Fig. 5. We would like to mention that results in Fig. 7 are for various Péclet number but the presentation is different in order to emphasis on the effect of gap width *b* which is the main factor of exploration in this work. We have mentioned various values of *b* in the legend of Fig. 7 and the values of Péclet number corresponding to these values of *b* are given in Table 2. An increase in interfacial length with an increase in Péclet number is evident and is in agreement with existing results that instability becomes more rigorous with an increase in $Pe_{num}$. But the important finding of our work is visualised on comparing the dynamics for same $Pe_{num}$ but different *b*. The interfacial length curve for $b=0.5$ mm in Fig. 8 and that for $b=1$ mm in Fig. 7 correspond to same value of $Pe_{num}$. It is evident that at all time, $0< L(t) < 250$ for the smaller value of *b* which is more than the *L*(*t*) value for $b=1$ mm for which $0<L(t) < 170$, $\forall t$. Thus, the VF instability is significantly influenced by the gap width. With an increase in *Pe*, instability becomes more rigorous but the effect of gap width is evident on the fingering pattern with no VF mechanism like tip splitting and shielding evident for same *Pe* but higher *b*.

### Comparison of the two dimensional and three dimensional results

Two dimensional studies of the flows through porous medium are conducted using Darcy’s law for the conservation of momemtum, coupled to equations governing the conservation of mass. Mathematically, the flow through a HS cell is comparable to the flow through a porous medium, and the flow is numerically modelled using Darcy’s law. However, as far as the authors’ know, no study focusses on the maximum allowed limit of *b* to neglect the three dimensional effects. Hence, we perform experiments for various gaps and mathematically model the flow using Stokes equation for conservation of momentum to take into account the three dimensional effects. As discussed in the previous section, the numerical simulations validate the experimental findings on the effect of gap on the VF dynamics. Here, we quantify the numerical results and compare with the two dimensional results obtained by modelling the flow using Darcy’s law. We cannot do simulations for $b < 0.24$ mm due to convergence issues. We can see in Fig. 8 that as we keep decreasing the gap, we approach the two-dimensional simulation results. This further shows that Stokes equation in a minimal gap HS cell gives similar results to Darcy’s law. Thus, the Stokes equation approaches the Darcy’s Law for small gap and the three dimensional effects can be neglected.

## Discussion

We aim to understand miscible VF for various gaps of the HS cell. The VF dynamics during radial displacement of miscible fluids in a HS cell are discussed. To investigate the impact of the gap on VF dynamics, several HS cell gap widths are taken into consideration. It is recognised that forces resulting from convection and diffusion compete and control the VF instability^2^. With an increase in the gap of the cell, the area between the glass plates increases. This results in an increased area available for the flowing fluid, decreasing the velocity of the flow on account of conservation of mass. Hence, a weaker convection is available resulting in a reduced instability. The instability is delayed with an increase in the gap of the cell which is in agreement with studies involving immiscible fluids wherein the instability is controlled by considering a tapered HS cell^24,25^ or an elastic membrane^26^ as top of the cell. We perform experiments to explore the extent of the gap up to which VF can be observed. The outcomes of the experiment are found to be in strong qualitative agreement with the carried out numerical study.

We vary the gap between the plates of the HS cell with the help of different spacers. It is observed that for a fixed log mobility ratio *M* and the injection rate *Q*, the increase in the gap results in a reduction of the instability. A rigorous instability with thin fingers undergoing merging, shielding and tip-splitting are observed for the smallest *b* considered. With variations in *b*, a transition in finger width is seen, with wider fingers appearing for the larger *b*. The VF dynamics are discussed using a non-dimensional parameter *Pe*. It is observed that the instability is enhanced with an increase in *Pe*. However, for a given fixed *Pe*, instability is weaker for the larger gap width indicating the effect of gap on instability. Increment in gap has a large effect on the overall dynamics of VF patterns due to a decrease in the convection. For the same radial distance away from the injection point, more volume is required to fill a larger gap. Since the volumetric flow rate is constant, this subsequently leads to a decrease in the effective convection of the displacing fluid. The larger the gap, the weaker the effective convection, and subsequently thicker the fingers in the patterns. Thus, proper care must be taken to design the cell to observe VF in the laboratory.

The experimental results are tested using non-linear simulations. A cylinder is considered to model a HS cell and the height of the cylinder works as the gap of the cell. The numerical results qualitatively agree with the experimental findings showing an abated instability with an increase in the gap. In addition, we model the flow using Darcy’s law and compare the results with the three dimensional simulations performed using Stokes equation for conservation of momentum. It is found that the Stokes results approach the Darcy results with a decrease in the gap.

The findings of this study will be helpful in modelling the flow through HS cell. The Hele–Shaw cell flow is used to model microfluidic devices^38^ and the effect of varying the gap on the flow dynamics reported in this work can help efficiently model the device depending upon the kind of dynamics required.

## Methods

### Experiment set-up

**Figure 9:**
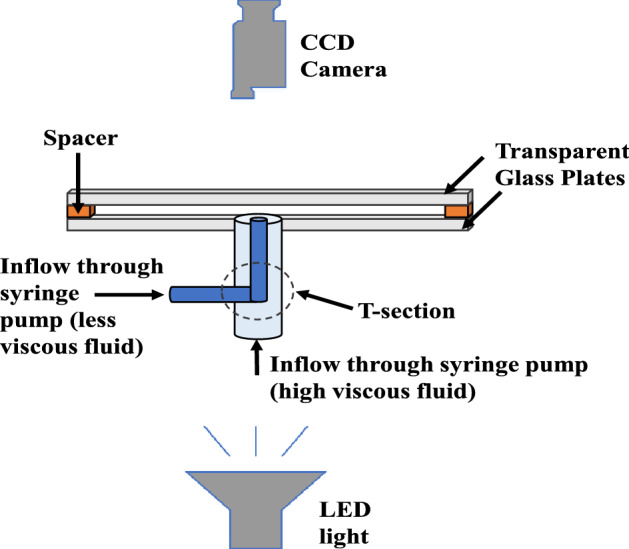
Hele–Shaw cell utilised in the experiments, shown schematically.

The experiments are conducted using a HS cell in which one fluid radially displaces the other. Two glass plates, each $300 ~\text {mm} \times 300 ~\text {mm} \times 10 ~\text {mm}$ in size, put parallel to one another with a very small space between them constitute the HS cell. The distance between the glass plates is denoted as *b* and is referred to as the width of the gap of the cell. The bottom plate has a hole for infusion of the high and the less viscous fluids. To conduct VF experiments, we need to inject less viscous and the more viscous fluids into the cell with the help of different injection pipes. Certain experimental errors like initial air trapping, sudden fluctuation in injection may happen during the change of pipes. Thus, to avoid the need to change the pipes for injection of different fluids and facilitate an effective infusion of fluids, we use a T-section^2^. The inner pipe of diameter 1.2 mm is embedded in the outer pipe with diameter 2 mm. The high viscous fluid is injected using the outer pipe while the inner pipe carries less viscous fluid. HS cell utilised is shown schematically in Fig. 9. The experiments are carried out by first injecting the high viscosity fluid into the cell, followed by a continuous injection of a less viscous fluid at a constant flow rate *Q* using a COLE-PARMER-D201253 pump. To evenly illuminate the HS cell and identify the interface, we combine LED backlighting with a paper diffuser. Dynamics are captured with the help of the Sony FDR-AX40 camera fixed above the cell.

#### Different gaps of the cell

The gap between the glass plates is obtained by placing spacers between the glass plates as shown in Fig. 9. To obtain various gaps in the experiments, we use four different spacers, $b = 0.3 ~\text {mm}, 0.5 ~\text {mm}, 0.8 ~\text {mm},$ and 1 mm. The difference in the gap is quite significant and some major challenges are faced while performing the experiments with large *b*. One of the challenge was to maintain uniform injection at large gaps. It is found that the existing cell is very good from the designing aspect for the gaps used in experiments which is limited to $b \ge 0.3 ~\text {mm}$ only.

#### Fluids used in the experimental study

Water and Glycerol are two miscible fluids used in the experimental examinations. Water is used as the less viscous fluid and different solutions of aqueous Glycerol serve as the more viscous fluids. Fluids having different viscosity are obtained by varying the concentration by volume of Glycerol in water. A highly purified distilled water is used both for the base fluid and the solvent for the higher viscosity fluids. For a visual contrast between the two fluids, Brilliant blue (BLENDS Ltd) food coloring at a concentration of 0.01 mg/ml^2^ is added to the less viscous fluid (distilled water). It is verified that the dye does not affect the viscosity of the fluid. A rheometer (Anton Paar, MCR 702) is used to investigate the viscosity of solutions in a cone and plate geometry at 25 $^{\circ }$C temperature. The log mobility ratio *M*, which is defined as the natural logarithm of the ratio of the viscosity of the displaced fluid (*µ*_2_) to that of the displacing fluid (*µ*_1_), that is $M=\ln ( \mu _2/\mu _1)$, is used to describe the viscosity contrast between the fluids. When there is no viscosity contrast between the fluids, then $M=0$. In our experiments, the displacing fluid is always less viscous and thus $M > 0$ as $\mu _1< \mu _2$. For $M=2$ , $\mu _2= 6.84 ~\text {mPa} \cdot$s, and $M=3$, $\mu _2=18.19 ~\text {mPa} \cdot$s while $\mu _1= 0.92 ~\text {mPa}\cdot$s.

#### Image consideration

The fingering patterns are captured using a camera (Sony FDR-AX40) with spatial resolution of 3840 $\times$ 2160 pixels and frame rate 25 fps. The camera is mounted with the center of the lens directly above the center of the HS cell. A light panel illuminates the cell from below, facilitating clear images. For visualization purpose, the displacing fluid (distilled water) is dyed. The intensity of color may differ in experiments for different gaps. Because the intensity of visible color correlates directly with the amount of the light passing through, the dyed fluid will appear lighter in smaller gap experiments than the larger *b*. Thus, the concentration of dye is chosen as 0.01 mg/ml so that the dyed fluid is easily distinguished from the defending fluid at the smallest gap thickness of 0.3 mm. The images are analyzed in ImageJ^39^ and MATLAB^40^ to monitor the interface’s location and determine its behaviour. Top view is shown in all figures showing experimental results. The experimental videos obtained from camera are processed with image processing tool ImageJ.

### Mathematical modelling

**Figure 10:**
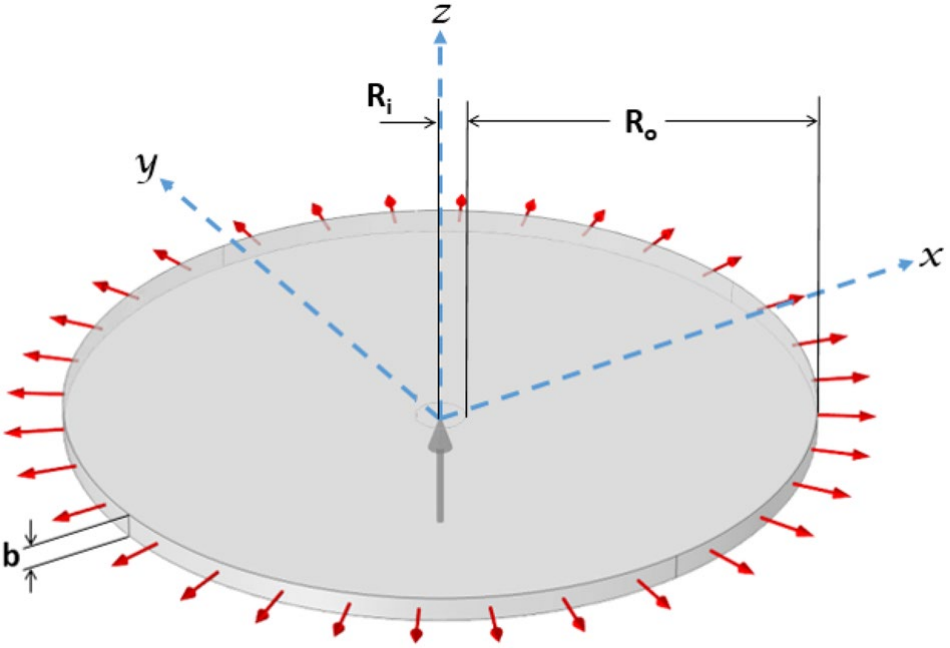
Schematic of a radial flow in a gap-width *b* radial HS cell. At the centre of the cell, a less viscous fluid is injected with a constant flow velocity U.

We consider HS cell with a gap thickness of *b* and two incompressible miscible viscous fluids. Following the terminology used in experiments, the fluid viscosities are indicated as *µ*_1_ (displacing fluid) and *µ*_2_ (displaced fluid), respectively. We model a HS cell by using a cylinder of radius $R_o$ and height *b*. To capture the fingering dynamics, $R_o$ is chosen sufficiently large. The height of the cylinder serves as the gap between the plates of the cell. A small hole with a radius of $R_i$ in the cylinder’s bottom is used to inject the less viscous fluid at a velocity of *U*. Figure 10 demostrates the basic geometry set up of our study with the injection hole depicted using a grey arrow. The centre of the injection hole serves as the origin of the Cartesian coordinate system (*x*, *y*, *z*). As shown by the red arrows in Fig. 10, the fluid is permitted to flow out from the geometry’s outside periphery. With the progress in time, the interface instability sets in and intricate dendritic patterns appear.

Previous work on VF is conducted using Darcy’s Law^41^ to capture the two dimensional porous medium flow. The Darcy’s Law (dl) module of COMSOL Multiphysics®^37,42^ is used to capture the dynamics. This module uses the continuity equation for conservation of mass and for momentum conservation, the Darcy’s Law is utilised. However, the three dimensional study cannot be performed using Darcy’s law. Hence, to understand and capture the three dimensional effects, the Stokes equation is used for the conservation of momentum. We use the Creeping Flow (*spf*) interface of COMSOL Multiphysics® to compute the velocity and pressure fields for the flow of fluids in the domain. The Stokes equation for momentum conservation and the continuity equation for mass conservation are the governing equations of our problem and are both solved by the Creeping Flow interface. Here, we solve the Stokes equation using the no-slip condition on the walls. Further, we have miscible fluids and an equation is required for the the mass conservation of the species. For this we use the Transport of Diluted Species in Porous Media (*tds*) interface of COMSOL Multiphysics®. This module defines the convection-diffusion equation for solute concentration and is utilized for studying the concentration evolution.

The dimensional governing equations for constant density $\rho$ can be written as^37^

3 $$\begin{aligned} \nabla \cdot \vec {u}= & {} 0, \end{aligned}$$

4 $$\begin{aligned} \frac{\partial \vec {u}}{\partial t}= & {} \nabla \cdot \Big [-p{{\textbf {I}}}+\mu \Big (\nabla \vec {u}+(\nabla \vec {u})^T\Big )\Big ], \end{aligned}$$

5 $$\begin{aligned} \varepsilon _p\frac{\partial c}{\partial t}+\vec {u}.\nabla c= & {} \varepsilon _pD\nabla ^2c, \end{aligned}$$

here, *p*, $\vec {u}$, and $\mu$ represent the dimensional pressure, velocity vector and viscosity, respectively. The porosity of the medium, which is a measurement of the fraction of void space, is represented by the constant $\varepsilon _p$. The fluid viscosity $\mu$ is assumed to be dependent on the concentration *c* as

6 $$\begin{aligned} \mu (c)= & {} \mu _1e^{Mc/c_{ref}}, \end{aligned}$$

where $M=\ln (\mu _2/\mu _1)$ is the log-mobility ratio and $c_{ref}=1\;\text {mol/m}^3$ is the reference concentration used to maintain dimensional homogeneity in the above equation. Initially only the more viscous fluid is contained within the domain (cell) and we inject the less viscous fluid from the injection hole at a constant velocity *U*. We set $c=0\;\text {mol/m}^3$ and $c=1\;\text {mol/m}^3$, respectively as the concentration of the injected (displacing) and the dispaced fluid. Thus, the initial condition is

7 $$\begin{aligned} \vec {u}\;(x,y,z,t=0)= & {} (0,0,U), ~at~ \; x^2+y^2 =R_i^2, \forall z, \nonumber \\ c\;(x,y,z,t=0)= & {} {\left\{ \begin{array}{ll} 0,&{} x^2+y^2 =R_i^2, \forall z\\ 1,&{} R_i^2<x^2+y^2 \le R_o^2, \forall z \end{array}\right. }, \end{aligned}$$

where *U* is the normal inflow velocity. Also, the less viscous fluid is injected continuously from the injection hole at the constant injection velocity *U* and the fluids are allowed to flow freely outside the domain with back flow suppressed. This can be represented by the first two boundary conditions given below

8 $$\begin{aligned} \vec {u}(x,y,z,t)= & {} (0,0,U), ~at~ \;x^2+y^2 =R_i^2, \forall z, \nonumber \\ p(x,y,z,t)= & {} 0, ~at~ \; x^2+y^2 =R_o^2, \forall z, \nonumber \\ \vec {u}(x,y,z,t)= & {} 0, \frac{\partial c}{\partial x}=0,\frac{\partial c}{\partial y}=0, ~at~ \;z=b, \forall x,y, \nonumber \\ \vec {u}(x,y,z,t)= & {} 0, \frac{\partial c}{\partial x}=0,\frac{\partial c}{\partial y}=0, ~at~ \;z=0, R_i^2<x^2+y^2 \le R_o^2. \end{aligned}$$

The last two boundary conditions represent the no slip condition for velocity and no flux for concentration at the upper and lower rigid boundary of the cylinder.

#### Two way coupling

By creating a weak form of the governing equations, the coupled partial differential equations are solved using the finite element method in COMSOL Multiphysics®. We make use of the pre-built modules of COMSOL Multiphysics® to simulate the study. In the *spf* module, we define the flow as incompressible and do not include any turbulence model. The density of the fluids is taken as $1000\;\text {kg/m}^3$. The concentration *c* in the viscosity concentration relation is obtained from the *tds* module.

In the *tds* module, we define the velocity field as the velocity from the *spf* module. This couples the *tds* and the *spf* module. Thus, we have a two way coupling, wherein the concentration from *tds* is directed towards the viscosity in *spf* and the velocity field from *spf* is directed towards the *tds* module. The porosity of the medium is defined in the matrix properties. The parameters used to run the simulations are presented in Table 3.

**Table 3:** Parameters for simulation.

Symbols	Parameters	Value and units
$R_i$	Radius of injection hole	0.6 mm
$R_o$	Radius of the cylinder	120 mm
*b*	Gap thickness	0.24 mm, 0.3 mm, 0.5 mm,
		0.8 mm, 1.0 mm.
*U*	Injection velocity	$5 ~\text {mm/s}, 10 ~\text {mm/s}$,
$\mu _1$	Viscosity of displacing fluid	1 mPa$\cdot$ s
*M*	Log mobility ratio	2, 3
D	Diffusion coefficient	$2 \times 10^{-8} ~\text {m}^2/s$
$\varepsilon _p$	Porosity	0.5
$c_{ref}$	Reference concentration	1 mol/m$^3$

#### Meshing

The meshing of the domain is done using user-controlled mesh. We do not choose the default tetrahedral meshing as the degree of freedom (DOF) as well as the the computation time are very high with such meshing due to three dimensional nature of the study. Instead, we use a free triangular mesh, calibrated for fluid dynamics, in the lower boundary and swept it all the way upto the upper boundary. The swept distribution for the least gap was taken as 2 because of convergence issues at distribution equal to 1. As the gap was increased, the distribution was also increased with maximum being 5. The maximum and minimum element size was fixed as 0.144 mm and 0.048 mm respectively with the element growth rate as 1.1.

The final time of the numerical simulations is kept large enough to keep the injected displacing fluid within the cylinder borders. This ensures no breakthrough of the injected fluid. For time discretization, COMSOL Multiphysics® uses finite difference method to reduce computation time. Among the host of schemes that are available, we have used the backward differentiation formula (BDF) method, with adaptive time stepping. Although the direct solvers will use more memory than the iterative solvers, but they are more robust. Thus, the linear system of equations are solved using direct (not iterative) segregated solver PARDISO as it takes less computation time. The Table 3 shows various gaps used in the numerical study. In our case, we limited ourselves to $b = 0.24 ~\text{mm}$, as no proper meshing was obtained for $b < 0.24$ which led to convergence issues.

## Data Availability

All data generated or analysed during this study are included in this published article.

## Abbreviations

VF: Viscous fingering
HS: Hele–Shaw
